# Generation and analysis of whole-genome sequencing data in human mammary epithelial cells

**DOI:** 10.5808/gi.22044

**Published:** 2023-03-31

**Authors:** Jong-Lyul Park, Jae-Yoon Kim, Seon-Young Kim, Yong Sun Lee

**Affiliations:** 1 Personalized Genomic Medicine Research Center, KRIBB, Daejeon 34141, Korea; 2 Department of Functional Genomics, University of Science and Technology, Daejeon 34113, Korea; 3 Department of Cancer Biomedical Science, Graduate School of Cancer Science and Policy, National Cancer Center, Goyang 10408, Korea

**Keywords:** breast cancer, DNA variant, human mammary epithelial cells, whole-genome sequencing

## Abstract

Breast cancer is the most common cancer worldwide, and advanced breast cancer with metastases is incurable mainly with currently available therapies. Therefore, it is essential to understand molecular characteristics during the progression of breast carcinogenesis. Here, we report a dataset of whole genomes from the human mammary epithelial cell system derived from a reduction mammoplasty specimen. This system comprises pre-stasis 184D cells, considered normal, and seven cell lines along cancer progression series that are immortalized or additionally acquired anchorage-independent growth. Our analysis of the whole-genome sequencing (WGS) data indicates that those seven cancer progression series cells have somatic mutations whose number ranges from 8,393 to 39,564 (with an average of 30,591) compared to 184D cells. These WGS data and our mutation analysis will provide helpful information to identify driver mutations and elucidate molecular mechanisms for breast carcinogenesis.

## Introduction

**Fig. 1.**
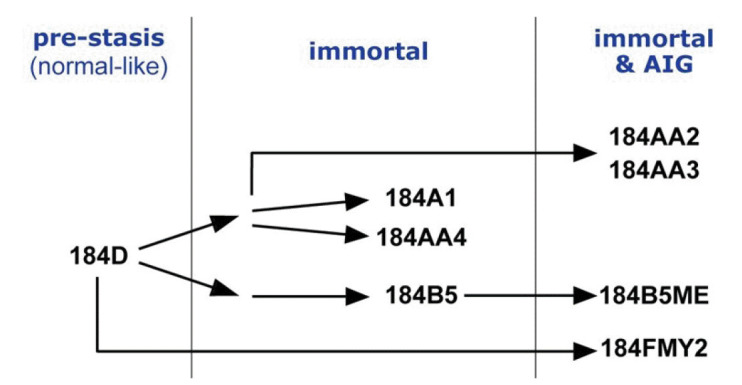
Diagram illustrating the human mammary epithelial cell progression series derived from a reduction mammoplasty specimen 184.

Breast cancer is the most common cancer diagnosed among women in the United States (excluding skin cancers). It is the second leading cause of cancer death among women after lung cancer [1]. It is curable in ~70%–80% of early-stage patients before metastasis. However, advanced breast cancer with distant organ metastases is considered incurable with currently available therapies [2]. Therefore, it is crucial to understand molecular characteristics that are associated with the development of breast cancer and to identify molecular biomarkers. A cell-based model system is essential for an in-depth study of molecular events during the human breast tumorigenesis. Human mammary epithelial cell (HMEC) lines, developed from normal breast tissues, are an ideal *in vitro* cell line model recapitulating early events of breast tumorigenesis [3] (see also https://hmec.lbl.gov/mock/history.html). Briefly, 184D is primary culture cells obtained from the reduction mammoplasty specimen 184. Most 184D cells underwent cell death (so-called the stasis barrier). 184D cells were treated with a mutagen benzo[a]pyrene or transformed by c-MYC transduction to overcome the stasis barrier. They were clonally selected to yield seven HMEC lines ([4-8], summarized in Fig. 1). In this study, we obtained and analyzed whole-genome sequencing (WGS) data from HMEC lines, which will help understand early breast carcinogenesis at the genomic level.

## Methods

### Cell line and other reagents

HMEC cultures were derived and grown as previously published [5-7,9]. The sources of other reagents were described in our previous study [10].

### WGS library construction and sequencing

We used the QIAamp DNA Mini Kit (Qiagen, Carlsbad, CA, USA) to isolate gDNA from HMEC cultures. The quantity of the extracted gDNA was analyzed with an ND-1000 spectrophotometer (Thermo Fisher Scientific, Waltham, MA, USA). For WGS library construction, we used the TruSeq DNA library Prep Kit (Illumina, San Diego, CA, USA) according to the manufacturer’s instructions. For WGS, paired-end sequencing was performed on the Illumina HiSeq X Ten sequencing instrument, yielding ~150-bp short sequencing reads.

### Data analysis

Raw sequence reads were aligned to the human reference genome 19 using Burrows Wheelers Aligner [11], and duplicate reads were removed using Picard (Broad Institute). We used Qualimap 2 to evaluate next-generation sequencing alignment data [12]. Then, the remaining reads were calibrated and realigned using the Genome Analysis Toolkit [13]. The realigned Binary Alignment Map files were analyzed using Strelka2 [14] to detect somatic single-nucleotide variants and insertions/deletions. The relative distribution of single-base substitutions was analyzed by the Maftools [15]. We used HOMER to annotate somatic mutation to the hg19 genome [16]. For driver mutation analysis, we download the driver gene list from the IntOgen cancer mutation browser [17]. For all programs, we used the default parameter setting.

### Data availability

The whole-genome data are available in the Korean Nucleotide Archive (KoNA, https://kobic.re.kr/kona) and Sequence Read Archive (SRA, https://www.ncbi.nlm.nih.gov/sra) public database with the accession number PRJKA220370 and PRJNA913438.

## Results and Discussion

### Quality and quantity of the sequencing data

We performed WGS on a total of eight HMEC cultures (shown in Fig. 1): pre-stasis 184D, its derivatives immortalized cell lines (184A1, 184AA4, and 184B5), and immortalized ones that further acquired AIG (184AA2, 184AA3, 184B5ME and 184FMY2). First, we assessed the quality and quantity of the WGS data, including mapping rates, genome coverage, scores of the mapping quality, and duplicate reads using Qualimap 2. These values are summarized in Table 1. Briefly, the mapping rate and scores of the mapping quality of the eight samples were higher than 85% and 53%, respectively. In addition, the average genome coverage was more than 30× (between 31.84× and 42.84×) in all eight samples. WGS data with 30× sequence coverage is appropriate for comprehensively identifying tumor-specific somatic mutations [18]. These results indicate that the quality and quantity of our WGS data were satisfactory for mutational analysis in HMEC cultures.

### Mutation patterns identified from the HMEC model

We analyzed somatic mutations from the WGS data. 184D cells are the primary culture of normal breast tissue and yet-to-be immortalized. Therefore, we considered 184D as normal breast tissue and used its genome sequence as a reference sequence when analyzing WGS data of the other seven HMEC lines that are cancer progression series.

**Fig. 2.**
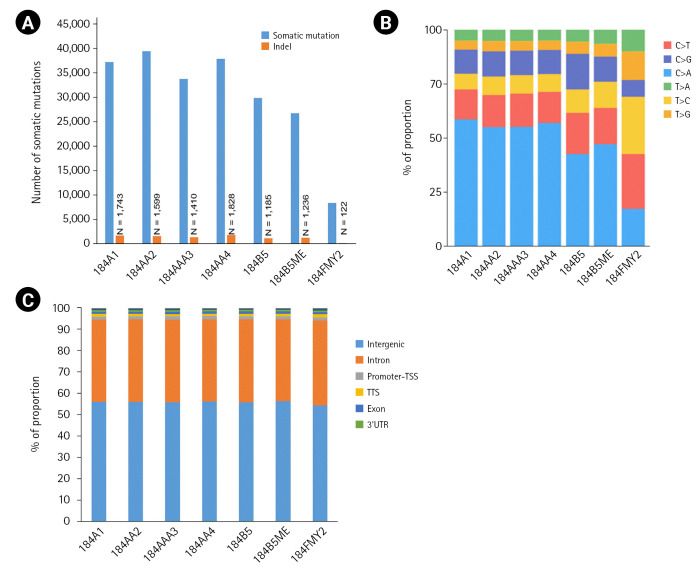
The number of somatic mutations and distribution of mutation types. (A) Somatic mutations were detected using the Strelka2 package with the default parameter setting. (B) Relative distribution of single-base substitutions by type in each human mammary epithelial cell culture sample. (C) Distribution of somatic mutation in the genome. Somatic mutations were annotated to the hg19 using the HOMER package. UTR, untranslated region.

Among the seven HMEC lines, the number of somatic mutations per sample ranged from 8,393 to 39,564, with an average of 30,591 (Fig. 2A). In particular, 184FMY2 had notably low somatic mutation frequency (n = 8,393), in agreement with the fact that it had been made by c-MYC transduction, whereas the other HMEC lines were treated with benzo[a]pyrene. Next, we examined the pattern of base substitutions. Except for 184FMY2, we observed that ~50% of mutations were C>A and that ~30% were C>T and C>G transversions (Fig. 2B), similarly to a previous study [19]. We annotated those somatic mutations to the hg19 reference genome and observed that most somatic mutations were located in the intergenic and intronic regions (Fig. 2C).

Since non-synonymous mutations are likely to be essential and are functionally annotatable, we focused on them. The number of mutations affecting protein-coding genes was 52 to 361 in each sample (data not shown). Then, we performed the driver mutation analysis using the IntOGen cancer mutation browser [17] and observed that 36 non-synonymous mutations in the HMEC cancer progression series coincided with the cancer driver mutations (Table 2). Further study will be needed to validate whether the mutated genes are genuinely associated with breast carcinogenesis.

In this study, we generated WGS data and analyzed mutation profiles in the HMEC cancer progression series because genetic mutations are one of the most significant factors in determining breast cancer progression and therapeutic management [20]. We hope that our WGS data of HMEC lines will provide useful information to breast cancer researchers and clinicians.

## Figures and Tables

**Table 1.** Quality and quantity of the sequencing data

Sample ID	Total No. of reads	Mapped reads, n (%)	Duplicate reads, n (%)	Genome coverage (mean)	Mapping quality
184A1	913,043,618	853,249,715 (93.45)	118,589,417 (12.99)	40.62	54.03
184AA2	870,033,090	826,770,914 (95.03)	101,833,624 (11.7)	39.31	53.90
184AA3	819,634,530	733,795,759 (89.53)	95,136,431 (11.61)	34.95	53.98
184AA4	794,654,212	762,287,813 (95.93)	102,740,630 (12.93)	36.31	53.98
184B5	1,008,170,478	898,909,936 (89.16)	164,770,658 (16.34)	42.84	53.96
184B5ME	799,810,062	734,277,857 (91.81)	92,112,929 (11.52)	35.00	54.08
184D	779,003,944	669,465,976 (85.94)	86,326,513 (11.08)	31.87	54.00
184FMY2	828,214,280	791,321,104 (95.55)	117,711,972 (14.21)	37.69	54.02

**Table 2.** Annotation of non-synonymous mutations in each of HMEC samples to a cancer driver mutations database

Symbol	Immortal			Immortal with AIG			
	184A1	184AA4	184B5	184AA2	184AA3	185B5ME	184FMY2
*MTOR*	Q1627K	Q1627K	ND	Q1627K	Q1627K	ND	ND
*CSF3R*	T154N	T154N	ND	T154N	T154N	ND	ND
*KMT2D*	M1417I	M1417I	ND	M1417I	M1417I	ND	ND
*ACSL3*	G476V	G476V	ND	G476V	G476V	ND	ND
*PLCG1*	D342Y	D342Y	ND	D342Y	D342Y	ND	ND
*CARD11*	T43M	T43M	ND	T43M	T43M	ND	ND
*AMER1*	P49Q, P49T	P49Q, P49T	ND	P49Q, P49T	P49Q, P49T	ND	ND
*BTK*	R332S	R332S	ND	R332S	R332S	ND	ND
*NFKBIE*	K316N	ND	ND	K316N	K316N	ND	ND
*SETBP1*	D412H	D412H	ND	D412H	ND	ND	ND
*CDKN2A*	G102V	G102V	ND	G102V	ND	ND	ND
*MED12*	M880I	M880I	ND	ND	M880I	ND	ND
*RSPH10B2*	S71T	ND	ND	ND	S71T	S71T	ND
*FOXD4L1*	ND	ND	P234R	Y110S, P234R	ND	Y110S	ND
*CIITA*	ND	ND	Q444K	ND	ND	Q444K	ND
*ZNF626*	ND	ND	G253R	ND	ND	G253R	ND
*CUL3*	ND	ND	G283V	ND	ND	G283V	ND
*MYH9*	ND	ND	L171F	ND	ND	L171F	ND
*FGD5*	ND	ND	G395C	ND	ND	G395C	ND
*NPRL2*	ND	ND	A141S	ND	ND	A141S	ND
*TET2*	ND	ND	E1144V	ND	ND	E1144V	ND
*ABCB1*	ND	ND	R905G	ND	ND	R905G	ND
*CDH11*	ND	R218I	ND	R28Q	ND	ND	ND
*PDPR*	ND	ND	ND	ND	ND	I47V	I47V
*H3F3A*	A115G	ND	ND	ND	ND	ND	ND
*CIC*	T1560P	ND	ND	ND	ND	ND	ND
*NOTCH2*	ND	ND	P210L	ND	ND	ND	ND
*PEG3*	ND	Q1182H	ND	ND	ND	ND	ND
*KDM3B*	ND	ND	N1092Y	ND	ND	ND	ND
*LRP1B*	ND	ND	G838R	ND	ND	ND	ND
*PML*	ND	ND	L825V	ND	ND	ND	ND
*CLTCL1*	ND	ND	E1304Q	ND	ND	ND	ND
*RHPN2*	ND	ND	ND	ND	K216R	ND	ND
*FANCD2*	ND	ND	ND	ND	P87R	ND	ND
*ZNF429*	ND	ND	ND	ND	ND	ND	S498R

HMEC, human mammary epithelial cell; ND, not detected.
